# Metabolic syndrome among a middle-aged population in the Red River Delta region of Vietnam

**DOI:** 10.1186/1472-6823-14-77

**Published:** 2014-09-26

**Authors:** Tran Quang Binh, Pham Tran Phuong, Bui Thi Nhung, Do Dinh Tung

**Affiliations:** 1 National Institute of Hygiene and Epidemiology, 1 Yersin, Hanoi, Vietnam; 2 Dinh Tien Hoang Institute of Medicine, 20 Cat Linh, Dong Da, Ha Noi, Vietnam; 3 National Institute of Nutrition, 48B Tang Bat Ho Street, Hanoi, Vietnam; 4 National Institute of Diabetes and Metabolic Disorders, 1 Ton That Tung, Hanoi, Vietnam

**Keywords:** Metabolic syndrome, Population–based study, Prevalence, Associated factors, Vietnamese

## Abstract

**Background:**

Metabolic syndrome (MetS) is a clustering of metabolic risk factors for cardiovascular diseases and type 2 diabetes. The study aimed to estimate the prevalence of MetS, its components, and their associations among rural middle-aged population in Vietnam.

**Methods:**

A cross-sectional study with a representative sample (n = 2443) was conducted to collect data on demographic, socioeconomic, anthropometric, lifestyles, plasma glucose, and lipid profile. The age- and sex-adjusted prevalences of MetS and its components were calculated using the direct standardization. Associations of risk factors with MetS were evaluated using logistic regression, taken into account the confounding factors.

**Results:**

The total age- and sex-adjusted prevalence (95% CI) of MetS was 16.3% (14.0 - 18.6). The most frequent component of MetS was high triglycerides (43.2%), followed by low HDL-C (42.0%), elevated blood pressure (29.2%), high plasma glucose (14.3%), and central obesity (12.3%). Of the total population, only 17.6% did not have any component of MetS and more than 40% had at least two MetS components. The association of MetS with residence, age, body mass index, marital status, and siesta time per day was statistically significant in univariate analysis and replicated in multivariate analysis.

**Conclusion:**

The MetS prevalence and its components are common and major public health burden in the middle-aged adults in Vietnam. Habitants living in urban, being never-married, having an increase in age, BMI, and siesta time per day are significantly associated with MetS, and they should be paid much more attention for screening and implementing preventive activities.

## Background

Metabolic syndrome (MetS) is a clustering of metabolic risk factors including central obesity, elevated blood pressure, increased fasting plasma glucose, high serum triglycerides, and low high-density cholesterol levels [1]. People with metabolic syndrome are at increased risk for atherosclerosis, peripheral vascular disease, coronary heart disease, myocardial infarction, stroke, and type 2 diabetes [2-5], which are the leading causes of death and disability worldwide [6]. However, metabolic syndrome and its deadly consequences can be preventable and treated by maintaining a healthy weight, eating a healthy diet, getting adequate physical activity, and following healthcare providers’ instructions [7,8]. To prevent from premature deaths and illnesses, it is necessary to evaluate the magnitude of metabolic risk factors and identify population groups at risk of chronic diseases.

Despite the worldwide importance of MetS, relatively little has been known about its actual prevalence and its risk factors in Vietnam. According to the US National Cholesterol Education Adult Treatment Panel III (NCEP ATPIII) criteria [9], the prevalence of MetS in Ho Chi Minh city, the biggest city in the Southern Vietnam, was 12% in adults aged ≥ 20 years in 2001 [10], and 8.2% population aged ≥ 15 years in 2003 suffered MetS in Khanh Hoa, a coastal province of South Central Vietnam [11]. The MetS prevalence was found in 4.6% children aged 13–16 years in Ho Chi Minh City in 2007 [12]. To date, there has been a limited data on the MetS and associated factors in rural areas with more than 71% of the total population in Vietnam [13]. In addition, a better knowledge of the components of MetS should provide important insights in the pathogenesis of MetS, allowing the evaluation of better interventions at both population level and individual level to reduce the burden of MetS. Therefore, we conducted a cross–sectional study to identify the prevalence of MetS and its components, as well as associated factors for MetS in rural Vietnamese population.

## Methods

### Study design and subjects

Located in the western coastal zone of the Gulf of Tonkin, the Red River Delta region is the flat plain formed by the Red River and the Thai Binh River in northern Vietnam. It is an agriculturally rich area and densely populated. Out of 85.8 million Vietnam people, are over 19.6 million live in the Red River region including 10 provinces: Bac Ninh, Hai Duong, Hung Yen, Hai Phong, Hanoi, Vinh Phuc, Ha Nam, Thai Binh, Nam Dinh, and Ninh Binh. Ha Nam province is a typical rural province in the south–west of the Red River Delta region and 50 km far from Hanoi >Capital, Vietnam. Its population has approximately 800,000 inhabitants, living mostly in rural areas (108 rural communes and 6 urban wards) [12]. To investigate the magnitude of chronic and non-communicable diseases, a cross–sectional study was designed and conducted in Ha Nam province from July to November 2011. The Ethics Committee of the National Institute of Hygiene and Epidemiology, Vietnam approved the protocol of the survey. All participants provided written informed consent before entering the study.

A representative sample size of Ha Nam habitants aged 40–64 years was estimated and recruited randomly using the two–stage sampling method. First, 30 communes and wards were selected from all 114 communes and wards in Ha Nam province, using the probability proportion–to–size method. From the list of all local persons aged 40–64 years in each of the selected communes and wards, 100 unrelated participants were recruited using the simple random sampling method. The principle of simple random sampling is that every object has the same probability of being chosen. Exclusion criteria for potential participants included pregnant women, critically ill subjects, and mentally disordered subjects. In the days of survey, 290 (9.7%) subjects were absent or excluded due to ages < 40 or > 64 years. Of the 2710 participants took part into the survey, 2443 people with complete data on five components of MetS were included in the analysis for the present study.

### Data collection

We conducted a survey to collect data. Details of the survey were reported previously [14]. All surveyors were trained to use the questionnaire and they practiced interviewing local people in the field. The questionnaire was validated in this stage. The trained surveyors interviewed participants to complete a structured questionnaire. Data were collected on current age, ethnicity, educational level, occupation, family history of diabetes, history of hypertension and dyslipidemia, medical and reproductive history, smoking and drinking history, time spent for night’s sleep, siesta, and watching television (TV). Usual alcohol consumption over the past year was assessed via an in–person interview. For each beverage type (beer, wine), participants reported their consumption frequency and portion size. These data were combined to yield 4 alcohol consumption categories: none (never or < 1 drink/mo), ≥ 1 drink/mo to < 1 drink/wk, 1 drink/wk to ≤ 1 drink/d, and ≥ 2 drink/d, in which one drink was defined as a 50–ml cup of rice wine at about 30%. The surveyors also measured anthropometrics including weight, height, waist circumference (WC), hip circumference (HC), and percent body fat. Body fat percentage was measured by bioelectrical impedance method by using OMRON scale (HBF–351, Kyoto, Japan). Systolic blood pressure (SBP) and and diastolic blood pressure (DBP) were measured twice in a sitting position after participants rested for at least 5 min. The mean of the two values was used in the analysis. Blood samples were collected and centrifuged immediately in the morning after a participant had fasted for at least 8 h prior to the clinic visit. Aliquots of plasma were stored at 2–8°C in iceboxes and then transported into the Biochemistry Laboratory of the Ha Nam Center for Preventive Medicine for analysis within 6 hours. Plasma glucose was measured by glucose oxidase method (GOD–PAP). Lipid profile including total cholesterol (TC), triglycerides (TG), high-density lipoprotein cholesterol (HDL-C), and low-density lipoprotein cholesterol (LDL-C) were measured by enzymatic methods. Glucose and lipid profile were analyzed using a semi–autoanalyzer (Screen Master Lab; Hospitex Diagnostics LIHD112, Italy) with commercial kit (Chema. Diagnostica, Italy).

Metabolic syndrome was classified according to the consensus criteria endorsed by the US National Cholesterol Education Adult Treatment Panel III (NCEP ATPIII), the International Diabetes Federation (IDF), and several other organizations [15]. It is the modified ATP III criteria, adjusted for waist circumference in Asian population, as suggested by the IDF definition. A participant was classified as having MetS as the presence of three or more the following: 1) fasting plasma glucose ≥ 100 mg/dL (5.6 mmol/L) or diabetes; 2) systolic blood pressure ≥ 130 mmHg or diastolic blood pressure ≥85 mmHg or hypertension; 3) HDL-C < 40 mg/dL (1.04 mmol/L) for men and HDL-C < 50 mg/dL (1.29 mmol/L) for women; 4) Triglycerides ≥ 150 mg/dL (1.7 mmol/L); 5) Waist circumference ≥ 90 cm for men and ≥ 80 cm for women.

### Statistical analysis

Data were weighted, taken into account the study design, the probability of sampling, finite population correction, and none–response rate. The estimated prevalences of MetS and its components (central obesity, elevated blood pressure, high triglycerides, low HDL-C, and increased fasting glucose) were calculated for the whole Ha Nam population aged 40–64 years together with subgroups according to age group, sex, nutritional status, and socioeconomic status. The age and sex–adjusted prevalences were estimated using direct standardization method based on the 2009 Vietnam Population and Housing Census [13].

Independent–Sample *T* test or Mann–Whitney *U* test was used appropriately to compare continuous variables between women and men after checking for normal distribution. Pearson’s *χ*^2^ test or Fisher’s exact test was performed to compare frequencies of category variables. Univariate logistic regression analysis was used to assess potential factors associated with MetS. Multivariate logistic regression analysis was performed to test the associations of MetS to the potential associated factors including: i) socioeconomic conditions: age, sex, residence, marital status, income level, occupation, and educational levels; and ii) lifestyle - related factors: alcohol consumption, smoking, time spending for night’s sleep, siesta, sitting, and watching TV. Data are expressed as odds ratios with 95 percent confidence intervals (CI). Two–sided *P* < 0.05 was considered statistically significant. The above statistical procedures were performed using Stata version 11.2 and SPSS version 16.0.

## Results

### Characteristics of the studied cohort

Of the 2443 participants with complete data of MetS components, 1594 (65.2%) were women, 1743 (71.3%) were famers, 1760 (72%) had primary or secondary education, 631 (25.2% in men and 0.3% in women) were tobacco smokers, and 852 (27.3% in men and 7.6% in women) were alcohol drinkers. The characteristics of the subjects by gender are shown in Table 1. Median age (interquartile range) was 52 (48–57) years in men and 51 (45.4-56) years in women. BMI and lipid profile (TG, TC, HDL-C, and LDL-C) were not statistically different between men and women. Age, anthropometric measures (height, weight, WC, HC, WHR, SBP, and DBP), and fasting plasma glucose were significantly higher in men compared to women, while percent body fat was much lower in men than in women.

**Table 1:** Characteristics of the studied subjects by gender

**Variables**	**Women**	**Men**	*P*-value*
	**(*n *** **=1594)**	**(*n*** **= 849)**	
Age (year)^†^	51.0 (45.4 - 56.0)	52 (48–57)	< 0.0001
Height (cm)	152.2 ± 5.0	162.0 ± 5.3	< 0.0001
Weight (kg)	49.7 ± 7.0	56.1 ± 8.1	< 0.0001
BMI (kg/m^2^)	21.4 ± 2.6	21.4 ± 2.7	0.661
Body fat (%)	30.2 ± 4.6	22.1 ± 5.4	< 0.0001
WC (cm)^†^	73 (68–78)	76.8 (70.5 - 82.5)	< 0.0001
HC (cm)^†^	87.6 (84–91)	89 (85–92)	< 0.0001
WHR†	0.83 (0.79 - 0.87)	0.86 (0.82 - 0.90)	< 0.0001
SBP (mmHg)^†^	110 (100–125)	120 (110–130)	< 0.0001
DBP (mmHg)^†^	70 (60–80)	77.5 (70–80)	< 0.0001
Fasting plasma glucose^†^	4.6 (4.0 - 5.2)	4.7 (4.2 - 5.3)	0.003
Total cholesterol^†^	4.3 (3.9 - 4.9)	4.28 (3.9 - 4.94)	0.966
HDL-C^†^	1.23 (0.98 - 1.59)	1.20 (0.97 - 1.61)	0.511
LDL-C^†^	2.86 (2.37 - 3.42)	2.86 (2.35 - 3.43)	0.712
Triglycerides^†^	1.40 (1.01 - 2.10)	1.49 (1.01 - 2.26)	0.078

BMI, body mass index; WC, waist circumference; HC, hip circumference; WHR, waist–hip ratio; SBP, systolic blood pressure; DBP, diastolic blood pressure; HDL-C, high-density lipoprotein cholesterol; LDL-C, low-density lipoprotein cholesterol. Data are mean ± SD unless otherwise indicated.

^†^Data are median (interquartile range). *P-value: women *vs.* men by Student–*T* test or Mann–Whitney *U* test.

### Prevalence of metabolic syndrome and its components

Table 2 presents the estimated prevalences of MetS and its components among middle-aged population in Ha Nam province. The total age– and sex–adjusted prevalence (95% CI) of MetS was 16.3% (14.0 - 18.6). The adjusted prevalence of MetS increased with age and reached a peak at 60–64 age group. The most frequent component of MetS was high TG (43.2, 95%CI: 39.5 - 46.9%), followed by low HDL-C (42.0, 95%CI: 38.6 - 45.5), elevated blood pressure (29.2, 95%CI: 26.0 - 32.4), high plasma glucose (14.3, 95%CI: 12.1 - 16.6), and central obesity (12.3, 95%CI: 10.4 - 14.2). Prevalence of MetS, central obesity, and low HDL-C was remarkably higher in women compared to men, while prevalence of elevated blood pressure and high TG was much higher in men compared to women. Men had a trend of higher elevated fasting plasma glucose than women.

**Table 2:** The estimated prevalence of metabolic syndrome and its components among middle-aged population in Ha Nam province, 2011

**Age group**	**N**	**Metabolic syndrome**	**Central obesity**	**Elevated blood pressure**	**Increased blood glucose**	**High TG**	**Low HDL-C**
Total population							
40 - 44	446	7.9 (6.4 - 9.5)	7.3 (5.6 - 8.9)	17.1 (13.9 - 20.4)	12.8 (10.5 - 15.1)	34.8 (31.0 - 38.6)	38.8 (35.1 - 42.5)
45 - 49	584	12.3 (10.3- 4.3)	9.3 (7.8 - 10.8)	23.3 (20.5 - 26.0)	11.8 (9.8 - 13.8)	42.1 (38.6 - 45.7)	41.4 (38.0 - 44.7)
50 - 54	613	19.2 (16.8 - 21.5)	16.2 (14.3 - 18.2)	32.8 (29.4 - 36.1)	14.3 (12.2 - 16.4)	44.2 (40.4 - 47.9)	45.2 (41.9 - 48.4)
55 - 59	481	26.3 (23.4 - 29.2)	16.9 (14.7 - 19.1)	45.1 (41.0 - 49.2)	18.8 (16.3 - 21.3)	51.6 (48.0 - 55.1)	43.5 (40.0 - 47.0)
60 - 64	319	26.9 (23.1 - 30.8)	17.1 (14.7 - 20.0)	43.8 (40.7 - 46.9)	18.9 (15.9 - 21.9)	52.5 (48.9 - 56.2)	42.6 (38.9 - 46.4)
Adjusted total^†^	2443	16.3 (14.0 - 18.6)^c^	12.3(10.4 - 14.2)^c^	29.2(26.0 - 32.4)^c^	14.3 (12.1 - 16.6)^c^	43.2 (39.5 - 46.9)^c^	42.0 (38.6 - 45.5)
Women							
40 - 44	336	7.7 (6.3 - 9.0)	10.8 (9.0 - 12.5)	9.2 (7.6 - 10.8)	9.1 (7.6 - 10.6)	34.8 (31.5 - 38.1)	52.9 (48.8 - 57.0)
45 - 49	396	13.0 (11.1 - 14.9)	15.5 (13.5 - 17.6)	18.0 (15.5 - 20.6)	10.5 (8.7 - 12.3)	39.0 (35.9 - 42.1)	51.4 (48.0 - 54.9)
50 - 54	382	23.1 (20.4 - 25.8)	26.1 (23.5 - 28.8)	29.0 (25.5 - 32.5)	13.6 (11.5 - 15.8)	43.6 (39.9 - 47.3)	51.4 (48.3 - 54.5)
55 - 59	291	30.1 (27.1 - 33.0)	24.7 (22.0 - 27.4)	41.4 (37.0 - 45.8)	18.4 (15.7 - 21.0)	48.3 (45.1 - 51.5)	54.2 (50.8 - 57.5)
60 - 64	189	32.3 (28.6 - 36.0)	28.8 (25.1 - 32.5)	37.1 (34.2 - 40.0)	21.0 (18.0 - 23.9)	54.8 (51.4 - 58.1)	53.3 (49.7 - 56.8)
Adjusted total^†^		18.5 (16.3 - 20.7)^c^	19.5(17.2 - 21.8)^c^	23.8 (21.1 - 26.6)^c^	13.1 (11.1 - 15.1)^c^	42.0 (38.8 - 45.3)^c^	52.4(49.0 - 55.8)
Men							
40 - 44	110	8.3 (6.3 - 10.3)	3.5 (1.9 - 5.1)	25.6 (20.5 - 30.7)^c^	16.7 (13.5 - 20.0)	34.7 (30.1 - 39.2)^a^	23.7 (20.1 - 27.3)
45 - 49	188	11.5 (9.3 - 13.7)	2.5 (1.5 - 3.5)	29.0 (25.9 - 32.1)	13.2 (10.9 - 15.5)	45.5 (41.3 - 49.8)	30.4 (26.9 - 33.8)
50 - 54	231	14.9 (12.8 - 17.0)	5.5 (4.2 - 6.7)	36.9 (33.5 - 40.2)	15.0 (12.8 - 17.2)	44.8 (40.8 - 48.8)	38.3 (34.8 - 41.9)
55 - 59	190	21.8 (18.9 - 24.8)	7.6 (5.8 - 9.4)	49.4 (45.4 - 53.5)	19.2 (16.8 - 21.7)	55.5 (51.3 - 59.4)	30.9 (27.0 - 34.8)
60 - 64	130	21.1 (17.0 - 25.1)	4.8 (3.2 - 6.5)	51.2 (47.6 - 54.7)	16.6 (13.4 - 19.9)	50.1 (45.9 - 54.3)	30.9 (26.8 - 35.1)
Adjusted total^†^		13.9 (11.5 - 16.2)^b^	4.4 (3.0 - 5.8)	35.1(31.3 - 38.8)^c^	15.7 (13.1 - 18.3)	44.5 (40.3 - 48.6)^a^	30.6 (27.1 - 34.2)

TG, triglycerides; HDL - C, high - density lipoprotein cholesterol. Data are expressed as number (%, 95%CI). ^†^Age and sex adjustment based on the 2009 Vietnam Population and Housing Census using direct standardization method. ^a^*P* < 0.05; ^b^*P* < 0.01; ^c^*P* < 0.001 by Chisquare test or Fisher exact test: compare metabolic syndrome and its components among age groups.

**Figure 1:**
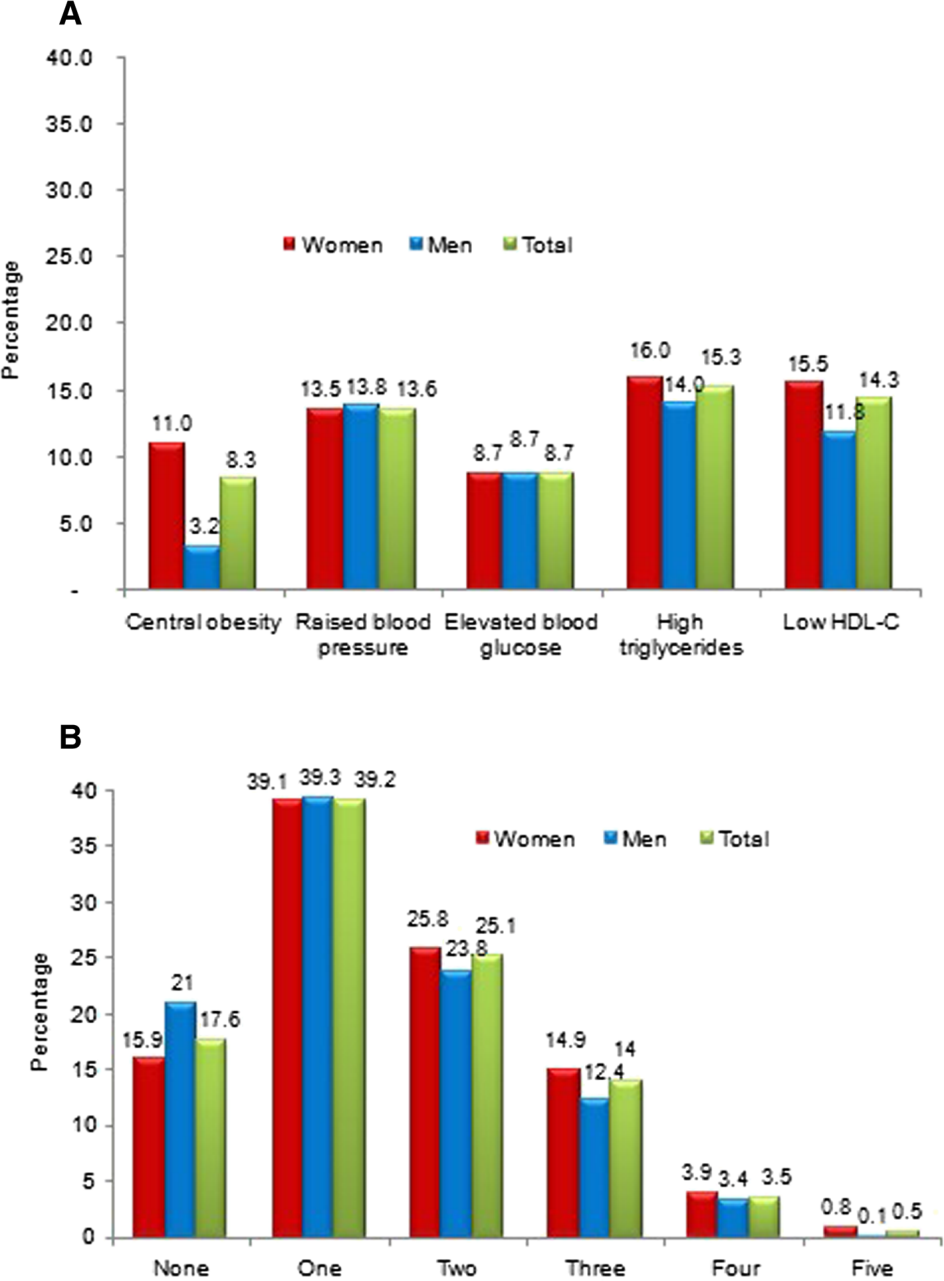
Prevalence of metabolic syndrome by: 1A) individual components and 1B) number of its components.

Prevalences of MetS and its components according to socioeconomic status and lifestyle patterns are presented in Additional file 1. There were significant differences of frequency of MetS according to residence, educational level, occupation, smoking, and nutrition status. There was no different distribution of MetS in levels of alcohol consumption, household incomes, marital status, and time spent for watching TV, sitting, siesta, and night’s sleeping. Except for low HDL-C, the other components of MetS were much higher in people living in urban area compared to rural area.In each group of central obesity, elevated blood pressure, increased plasma glucose, high TG, and low HDL-C, the MetS was found in 54.6, 44.2, 59.9, 35.0, and 30%, respectively. In the total cohort studied, the MetS prevalences in middle-aged people with central obesity, elevated blood pressure, increased plasma glucose, high TG, and low HDL-C, respectively, were 8.2, 13.3, 8.7, 14.6, and 14.1% (Figure 1A). The MetS prevalence according to number of its component is shown in Figure 1B. Adults without any component of MetS were found in 17.6% (21% in women, 15.9% in men) of the total sample. Adults with one, two, three, four, and five components of MetS accounted for 39.2, 25.1, 14, 3.5, and 0.5%, respectively.

### Associated factors for metabolic syndrome

Additional file 2 shows the potential association of each factor with MetS in univariate logistic regression analysis. Socioeconomic status (age, gender, educational level, and occupation), lifestyle patterns (residence, alcohol consumption, and smoking), anthropometric measures (BMI, PBF, HC, and WHR), and biochemical blood profile (TC and LDL-C) were significantly associated with MetS in univariate logistic regression. Further multivaritate logistic regression analysis of these potential associations of factors with MetS is presented in Table 3. The association of MetS with residence, age, BMI, marital status, and siesta time per day was found to be statistically significant in univariate analysis (Additional file 2) and replicated in multivariate analysis (Table 3).

**Table 3:** Associated factors of metabolic syndrome in multivariate logistic regression analysis

**Variable**	**Odds ratio (95%CI)**	***P***
Residence		
Rural	1.0	
Urban	2.46 (1.61–3.75)	< 0.0001
Gender		
Men	1.0	
Women	0.68 (0.43–1.09)	0.111
Age (year)	1.07 (1.05–1.09)	< 0.0001
Body mass index (kg/m^2^)	1.31 (1.25–1.37)	< 0.0001
Marital status		
Married	1.0	
Never	2.14 (1.06–4.31)	0.034
Widowed	0.96 (0.60–1.53)	0.868
Others	1.23 (0.49–3.14)	0.659
Education level		
Elementary	1.0	
Intermediate	0.71 (0.49–1.04)	0.075
Secondary	0.60 (0.36–1.02)	0.058
Post–secondary	0.83 (0.50–1.39)	0.487
Heavy occupation		
Yes	1.0	
No	0.95 (0.67–1.34	0.759
Income level		
< 25 percentiles	1.0	
25– < 50 percentiles	1.02 (0.73–1.42)	0.925
50– < 75 percentiles	1.04 (0.74–1.46)	0.836
≥75 percentiles	0.91 (0.64–1.29)	0.598
Alcohol consumption		
None	1.0	
<1 drink/mo	0.67 (0.38–1.16)	0.151
≥ 1 drink/mo to < 1 drink/wk	1.18 (0.70–1.98)	0.544
1 drink/wk to ≤ 1 drink/d	0.85 (0.52–1.40)	0.532
≥ 2 drink/d	1.29 (0.77–2.17)	0.338
Smoking		
None	1.0	
Current smoker	0.67 (0.38–1.13)	0.131
Ex–smoker	1.23 (0.75–2.04)	0.416
Watching TV/day		
≤ 3 hours	1.0	
> 3 hours	1.31 (0.79–2.16)	0.299
Sitting time/day		
≤ 4 hours	1.0	
> 4 hours	0.89 (0.69–1.14)	0.342
Sleeping time/day		
6-7 h	1.0	
<6 h	1.01 (0.74–1.40)	0.939
≥8 h	1.03 (0.77–1.37)	0.870
Siesta time/day (per 15 min)	1.08 (1.02–1.15)	0.015

Educational level was categorized in four groups, by number of years of schooling: elementary level (≤5 years), intermediate level (6–9 years), secondary level (10–12 years), and post–secondary level (>12 years). Occupation was categorized as heavy occupation (farmer and manual worker) or none heavy occupation (office clerks, teacher, retired worker, and house worker). One drink was defined as a 50–ml cup of rice wine at about 30%.

OR and *P* values were adjusted by all variables in the table.

## Discussion

People with metabolic syndrome are at high risk for developing type 2 diabetes and cardiovascular diseases. However, there have so far been few papers studying the MetS prevalence in Vietnam. The present study indicated that the total age– and sex–adjusted prevalence (95% CI) of MetS was 16.3% (14.0–18.6) in the middle-aged population in the Red River Delta region of Vietnam; and the prevalence of high TG, low HDL-C, elevated blood pressure, high plasma glucose, and central obesity were 43.2, 42.0, 29.2, 14.3, and 12.3%, respectively. In addition, because Ha Nam province is thought to be a typical rural province in Red River Delta Region with a population about 3727000 adults aged 40–64 years [13], we could estimate the number of residents with MetS, high TG, low HDL-C, elevated blood pressure, high plasma glucose, and central obesity are 607500, 1610000, 1565000, 1088000, 533000, and 458400, respectively. These estimated numbers can help to show the burden of MetS in the region and it is crucial for local health managers to make activity plan to control MetS and its components.

Using the NCEP ATPIII criteria adapted for Asians, this MetS prevalence (16.3% in 2009) in rural population aged 40–64 years seems to be lower than that (about 18.1% in 2001) in urban population aged 35–64 years in the biggest city [10] and higher than that (about 10% in 2003) in a coastal population aged over 35 years in the South Central Vietnam [11]. In comparison with other populations in Asia with the adapted NCEP ATPIII criteria, our study reported lower MetS prevalence than those reported in Malaysia [16], Eastern India [17], China [18], Indonesia [19], Philippine [20], Japan [15], and South Korean [21]. The MetS prevalence in this study was compatible with that in Bangkok, Thailand [22] and higher than that reported in Taiwan [23]. This difference could be explained by the traditional lifestyle may remain conservative in rural areas and by the “nutrition transition” [24] still keeps low stage in this process with 11% underweight subjects (BMI < 18.5 kg/m^2^) and 7.7% obese subjects (BMI ≥ 25 kg/m^2^) in the present study.

In this study, the most frequent component of MetS was high TG (43.2%), followed by low HDL-C (42.0%), elevated blood pressure (29.2%), high plasma glucose (14.3%), and central obesity (12.3%). In Vietnam, low HDL-C prevalence was reported to be the second-highest rate in rural area and another coastal area [11]. In addition, the contrast of central obesity prevalence was observed: lowest in rural and costal areas *vs.* highest in urban area [10]. Thus, MetS components vary in distribution in different geographical regions.

In terms of the subjects with normal BMI (18.5 ≤ BMI < 23 Kg/m^2^), the prevalences of MetS, central obesity, elevated blood pressure, increased plasma glucose, high TG, and low HDL-C were 12.5 (11.2 - 13.7), 4.3 (3.8 - 4.8), 26.5 (24.5 - 28.5), 12.9 (11.5 - 14.2), 42.6 (40.0 - 45.3), and 45.7% (43.0 - 48.4), respectively. Another study in the biggest city of Vietnam [25] showed that the rates of central obesity, hypertension, lipid metabolism disorders, and glucose metabolic disorders were 17.5, 29.3, 77.8 and 35.6%, respectively, in the subjects with normal BMI. The above data indicated that the prevalences of MetS and its components were relatively high even among those with BMI in normal ranges and at younger age.

With regard to the gender difference in MetS, we found that the prevalence of MetS, central obesity, and low HDL-C was remarkably higher in women compared to men, while prevalence of elevated blood pressure and high TG was much higher in men compared to women. It is in line with several studies in Vietnam [11], India [26], Korea [21,27], whereas it is different from other studies in ten large cohorts in European countries [28], and in Ghana [29]. This discrepancy can be explained by the different WC cut-off to define central obesity, age structure and characteristics of studied populations.

Predictors for an increased risk of developing MetS are very important to prevent a population from this disorder effectively. The present study indicated that residence, age, BMI, marital status, and siesta time per day were the most significantly associated factors for MetS. The association was found to be statistically significant in univariate analysis, replicated in multivariate analysis adjusted for socioeconomic conditions (age, sex, residence, marital status, income level, occupation, and educational levels), and lifestyle - related factors (alcohol consumption, smoking, time spending for night’s sleep, siesta, sitting, and watching TV). These associated factors should be validated in prospective studies for building prognosis models to early detection of MetS in rural Vietnamese populations, to warn people off high risk of MetS, and to counsel them how to prevent from MetS and its components.

Among the 5 Mets components, central obesity and elevated blood pressure are easy, non-invasive, and feasible criteria to use as the first step in the screening strategy for MetS detection in the context of developing countries with limited resources. In the present study, we used the waist circumference ≥ 90 cm for men and ≥ 80 cm for women to define central obesity [15]; and the prevalence of central obesity was found to be 12.3% (10.4 - 14.2), showing the lowest rate among the 5 MetS components. If screening strategy recruits only subjects with central obesity for further MetS detection (i.e., central obesity and any two of the others MetS components), 12.3% of the population is included in the second step, the sex- and age- adjusted prevalence of MetS is found 6.7% (5.4 - 8.0), indicating 5.6% underestimated prevalence or 54% undetected total MetS cases. If screening strategy uses elevated blood pressure as the first criteria (SBP ≥ 130 mmHg or DBP ≥85 mmHg), then 29.2% of the population with elevated blood pressure are recruited for the second step screening; and the adjusted MetS prevalence is 11.9% (9.9 - 13.9), showing 4.4% underestimated prevalence or 35% undetected total MetS cases. When central obesity or elevated blood pressure are used for the first step and second step using fasting blood analysis of glucose and lipid profile, 38.7% of the population is selected in the second step; and the adjusted MetS prevalence is 14.3% (12.0 - 16.5) with 2% underestimated prevalence or 12.3% undetected total MetS cases. This above analysis (Additional file 3) implicates that the better screening strategy to detect MetS in the Red River Delta region should include 2 steps, in which the first step is used for recruiting subjects with central obesity or elevated blood pressure, and second step is used for further analysis of glucose and lipid profile.

This study had several limitations. First, given the cross-sectional nature of the study, this does not allow for conclusions of the causal relationships. The follow-up study is needed to evaluate the cardiovascular events to estimate the incidence and develop the prognosis tools for early MetS detection. Second, data on physical activities and food intake were not used in these findings to evaluate a potential effect of these variables in our results. Lastly, our sample was a representative sample for a rural province in the Red River Delta region, the extrapolation for other geographical regions in Vietnam (mountainous, coastal, highland, and Mekong River Delta regions) should be taken into account. It is essential to conduct a national survey to evaluate the MetS patterns in different geographical regions.

## Conclusions

The study provides the general picture of MetS and its components in terms of prevalence and associated factors in the middle-aged population in the Red River Delta region, Vietnam. The MetS and its components are prevalent, even among those with BMI in normal ranges. More attention should be given to the elderly habitants living in urban, being never married, having an increase of age, BMI, and siesta time per day. Importantly, only 17.6% of the total population had not any MetS component and more than 40% had at least two MetS components. It highlights the urgent need for greater public awareness on prevalence and risk factors for MetS and strengthening of health services to detect, prevent, and treat early individuals with MetS.

## Abbreviations

MetS: Metabolic syndrome; BMI: Body mass index; WC: Waist circumference; HC: Hip circumference; WHR: Waist–hip ratio; SBP: Systolic blood pressure; DBP: Diastolic blood pressure; FPG: Fasting plasma glucose level; OGTT: Oral glucose tolerance test; TC: Total cholesterol, TG, Triglycerides, HDL-C, High-density lipoprotein cholesterol; LDL-C: Low-density lipoprotein cholesterol.

## Competing interests

The authors declare that they have no competing interests.

## Authors’ contributions

TQB: Conceptualization of the study, study design, proposal writing, data collection, data analysis, discussion and editing of the final draft for publication. PTP, BTN, DDT: Conceptualization of the study, study design, data collection, discussion and editing of the final draft for publication. All authors approved the final draft of this article prior to submission. All authors read and approved the final manuscript.

## Pre-publication history

The pre-publication history for this paper can be accessed here:

http://www.biomedcentral.com/1472-6823/14/77/prepub

## Supplementary Material

Additional file 1 **The estimated prevalence of metabolic syndrome and its components according socio - economic status and lifestyle factors among middle - aged population in Ha Nam province, 2011.** TG, triglycerides; HDL - C, high - density lipoprotein cholesterol. Data are expressed as number (%, 95%CI). Occupation was categorized as heavy occupation (farmer and manual worker) or none heavy occupation (office clerks, teacher, retired worker, and house worker). Overweight and obesity were defined as BMI ≥ 23 kg/m^2^ and BMI ≥ 25 kg/m^2^. One drink was defined as a 50–ml cup of rice wine at about 30%. †Age and sex adjustment based on the 2009 Vietnam Population and Housing Census using direct standardization method. ^a^*P* < 0.05; ^b^*P* < 0.01; ^c^*P* < 0.001 by Chisquare test or Fisher exact test: compare metabolic syndrome and its components among age groups. Click here for file

Additional file 2 **Associated factors of metabolic syndrome in middle-aged population in univariate logistic regression analysis.** LDL-C, low-density lipoprotein cholesterol; BMI, body mass index. Educational level was categorized in four groups, by number of years of schooling: elementary level (≤5 years), intermediate level (6–9 years), secondary level (10–12 years), and post–secondary level (>12 years). Occupation was categorized as heavy occupation (farmer and manual worker) or none heavy occupation (office clerks, teacher, retired worker, and house worker). One drink was defined as a 50–ml cup of rice wine at about 30%. High LDL-C was defined when LDL-C ≥ 130 mg/dL (≥3.4 mmol/L); High total cholesterol was defined when total cholesterol ≥ 200 mg/dL (≥5.2 mmol/L). Click here for file

Additional file 3 The proposal steps for screening metabolic syndrome in community. Click here for file
