# Supplementary material for: Metabolic syndrome among a middle-aged population in the Red River Delta region of Vietnam
Source: BMC Endocr Disord. 2014 Sep 26;14:77. doi: 10.1186/1472-6823-14-77 (PMC4179436; doi:10.1186/1472-6823-14-77)
Supplement: Additional file 1 — The estimated prevalence of metabolic syndrome and its components according socio - economic status and lifestyle factors among middle - aged population in Ha Nam province, 2011. TG, triglycerides; HDL - C, high - density lipoprotein cholesterol. Data are expressed as number (%, 95%CI). Occupation was categorized as heavy occupation (farmer and manual worker) or none heavy occupation (office clerks, teacher, retired worker, and house worker). Overweight and obesity were defined as BMI ≥ 23 kg/m2 and BMI ≥ 25 kg/m2. One drink was defined as a 50–ml cup of rice wine at about 30%. †Age and sex adjustment based on the 2009 Vietnam Population and Housing Census using direct standardization method. aP < 0.05; bP < 0.01; cP < 0.001 by Chisquare test or Fisher exact test: compare metabolic syndrome and its components among age groups. [file 1472-6823-14-77-S1.docx]

Additional file 1 − The estimated prevalence of metabolic syndrome and its components according socio−economic status and lifestyle factors among middle−aged population in Ha Nam province, 2011.

|  | **N** | **Metabolic syndrome** | **Central obesity** | **Elevated blood pressure** | **Increased blood glucose** | **High TG** | **Low HDL−C** |
| --- | --- | --- | --- | --- | --- | --- | --- |
| Nutritional status |  |  |  |  |  |  |  |
| Normal | 1467 | 12.5 (11.2−13.7)^c^ | 4.3 (3.8−4.8)^c^ | 26.5 (24.5−28.5)^c^ | 12.9 (11.5−14.2)^a^ | 42.6 (40.0−45.3)^c^ | 45.7 (43.0−48.4) |
| Overweight | 411 | 31.9 (29.1−34.7) | 36.9 (34.6−39.3) | 37.7 (34.5−40.8) | 18.4 (16.1−20.6) | 55.2 (51.5−58.9) | 44.5 (41.6−47.4) |
| Obesity | 229 | 44.0 (40.7−47.2) | 66.4 (63.0−69.8) | 48.9 (44.8−53.1) | 19.4 (16.3−22.5) | 53.8 (49.9−57.7) | 49.3 (46.2−52.4) |
| Underweight | 326 | 10.1 (8.0−12.3) | − | 22.4 (19.6−25.1) | 13.9 (11.9−15.9) | 30.7 (27.9−33.5) | 42.8 (39.8−45.8) |
| Marital status |  |  |  |  |  |  |  |
| Married | 2216 | 18.0 (16.5−19.5) | 14.9 (13.6−16.1) | 30.0 (27.9−32.1) | 14.1 (12.8−15.4)^a^ | 44.2 (41.6−46.8) | 45.0 (42.6−47.3) |
| Never | 49 | 28.7 (22.7−34.8) | 16.5 (10.9−22.1) | 42.1 (35.7−48.5) | 26.2 (20.5−31.9) | 38.9 (31.5−46.3) | 55.1 (50.1−60.2) |
| Widowed | 142 | 22.2 (19.5−24.9 | 18.4 (15.8−21.0) | 30.0 (26.2−33.8) | 17.3 (13.8−20.7) | 49.2 (44.3−54.1) | 46.6 (42.0−51.2) |
| Others | 36 | 16.8 (10.6−22.9) | 11.6 (7.1−16.0) | 17.1 (11.9−22.4) | 10.2 (5.5−14.9) | 36.4 (30.3−42.5) | 51.6 (42.5−60.8) |
| Education level |  |  |  |  |  |  |  |
| Elementary | 245 | 24.5 (22.3−26.8)^c^ | 17.6 (15.3.20.0)^a^ | 37.7 (33.8−41.6)^a^ | 14.1 (11.5−16.7) | 43.6 (40.3−46.8)^c^ | 49.5 (46.1−53.0) |
| Intermediate | 1515 | 16.9 (15.6−18.3) | 14.2 (12.9−15.6) | 28.4 (26.2−30.6) | 13.9 (12.5−15.2) | 42.3 (39.7−45.0) | 45.7 (43.1−48.3) |
| Secondary | 305 | 15.2 (12.9−17.4) | 11.9 (10.2−13.6) | 29.8 (26.9−32.7) | 13.4 (11.3−15.5) | 41.1 (38.2−43.9) | 42.6 (39.4−45.8) |
| Post–secondary | 378 | 23.0 (20.3−25.8) | 19.2 (17.1−21.3) | 31.9 (29.7−34.2) | 17.9 (15.3−20.6) | 54.8 (50.6−59.0) | 43.7 (40.6−46.9) |
| Residence |  |  |  |  |  |  |  |
| Rural | 2300 | 16.8 (15.6−17.9)^c^ | 14.1 (13.0−15.1)^c^ | 29.2 (27.0−31.4)^b^ | 13.3 (12.2−14.5) ^c^ | 41.5 (39.1−44.0) ^c^ | 46.2 (43.6−48.7) |
| Urban | 143 | 35.9 (30.3−41.6) | 25.6 (22.0−29.1) | 39.2 (36.9−41.6) | 26.6 (18.2−35.0) | 72.6 (71.3−73.9) | 37.1 (30.8−43.4) |
| Heavy occupation |  |  |  |  |  |  |  |
| Yes | 1929 | 16.7 (15.3−18.1)^c^ | 13.4 (12.2−14.6)^c^ | 29.0 (26.8−31.2) | 13.8 (12.4−15.1) | 41.3 (38.8−43.8) ^c^ | 45.9 (43.3−48.4) |
| No | 514 | 24.6 (22.2−27.0) | 20.8 (18.5−23.2) | 33.8 (31.6−36.1) | 17.1 (15.0−19.2) | 54.8 (51.1−58.4) | 43.6 (40.2−47.0) |
| Income level |  |  |  |  |  |  |  |
| < 25 percentiles | 598 | 16.0 (14.4−17.6) | 12.3 (10.7−13.9) | 27.8 (25.4−30.2) | 13.4 (11.9−14.9) | 42.1 (39.3−44.9) | 48.4 (45.0−51.7) |
| 25–<50 percentiles | 631 | 16.7 (15.2−18.3) | 14.4 (13.3−15.5) | 27.8 (25.3−30.3) | 13.6 (12.0−15.2) | 41.0 (37.7−44.3) | 44.6 (41.8−47.5) |
| 50–<75 percentiles | 587 | 18.9 (17.2−20.6) | 15.2 (13.7−16.7) | 31.2 (28.8−33.7) | 14.3 (12.5−16.2) | 44.7 (41.8−47.7) | 43.2 (40.6−45.8) |
| ≥75 percentiles | 627 | 22.1 (19.5−24.8) | 18.4 (16.6−20.3) | 33.4 (30.8−36.1) | 16.6 (14.7−18.6) | 49.2 (45.3−53.1) | 45.1 (42.1−48.1) |
| Alcohol consumption | |  |  |  |  |  |  |
| None | 1591 | 19.7 (18.1−21.3) | 18.8 (17.2−20.4)^c^ | 28.0 (25.7−30.3) ^c^ | 13.5 (12.0−15.1) | 43.3 (40.7−45.9) | 50.3 (47.6−52.9) ^c^ |
| <1 drink/mo | 135 | 14.7 (12.6−16.7) | 14.8 (12.4−17.2) | 20.5 (16.5−24.0) | 13.6 (11.0−16.2) | 44.7 (39.8−49.6) | 46.7 (42.7−50.7) |
| ≥ 1 drink/mo to < 1 drink/wk | 143 | 19.9 (16.3−23.5) | 10.9 (8.7−13.1) | 29.2 (25.7−32.6) | 18.0 (14.4−21.6) | 43.4 (38.6−48.2) | 45.7 (40.3−51.1) |
| 1 drink/wk to ≤ 1 drink/d | 316 | 12.9 (11.4−14.4) | 5.2 (4.2−6.1) | 34.2 (31.4−37.0) | 15.1 (13.2−17.0) | 43.7 (40.3−47.2) | 30.2 (27.4−32.9) |
| ≥ 2 drink/d | 258 | 18.3 (14.8−21.8) | 6.1 (4.9−7.3) | 43.4 (39.9−47.0) | 18.3 (15.5−21.2) | 51.3 (47.2−55.4) | 32.3 (29.1−35.5) |
| Smoking |  |  |  |  |  |  |  |
| None | 1812 | 19.6 (18.0−21.1)^c^ | 18.6 (17.1−20.1)^c^ | 27.0 (24.9−29.2)^c^ | 14.0 (12.5−15.4) | 43.1 (40.5−45.6)^c^ | 50.3 (47.7−52.9) ^c^ |
| Current smoker | 387 | 10.3 (8.8−11.9) | 2.7 (2.0−3.4) | 34.0 (31.4−36.7) | 14.6 (12.8−16.4) | 42.3 (38.5−46.1) | 28.5 (26.2−30.8) |
| Ex–smoker | 244 | 22.7 (19.4−26.0) | 8.0 (6.5−9.4) | 45.9 (42.0−49.7) | 18.1 (15.7−20.5) | 55.7 (51.8−59.5) | 35.3 (31.5−39.2) |
| Watching TV time/day |  |  |  |  |  |  |  |
| ≤ 3 hours | 2325 | 18.3 (16.8−19.7) | 15.3 (14.1−16.6) | 29.5 (27.4−31.6)^b^ | 14.3 (12.9−15.7) | 43.6 (41.0−46.3)^c^ | 45.4 (42.9−47.8) |
| > 3 hours | 118 | 22.0 (17.8−26.2) | 9.6 (7.7−11.5) | 40.7 (35.5−45.9) | 17.7 (14.1−21.3) | 56.5 (52.4−60.6) | 45.8 (41.1−50.5) |
| Siesta time/day |  |  |  |  |  |  |  |
| None | 356 | 19.3 (17.6−21.1) | 15.3 (13.8−16.8) | 30.5 (28.0−32.9)^a^ | 15.7 (13.6−17.7)^a^ | 43.7 (40.9−46.5) | 46.2 (43.2−49.2) |
| <30 min | 842 | 16.0 (13.7−18.3) | 16.1 (14.4−17.9) | 29.5 (26.3−32.6) | 12.3 (10.7−13.9) | 42.6 (39.3−45.8) | 45.7 (42.6−48.8) |
| 30–<60 min | 521 | 18.1 (16.3−19.9) | 12.4 (10.8−13.9) | 31.4 (28.4−34.4) | 15.8 (14.0−17.6) | 45.3 (42.4−48.1) | 44.1 (41.6−46.7) |
| 60–<90 min | 493 | 24.0 (21.1−26.9) | 17.9 (14.8−21.0) | 35.1 (31.5−38.6) | 18.2 (15.4−21.0) | 57.1 (52.6−61.5) | 39.0 (34.8−43.2) |
| ≥ 90 min | 168 | 31.2 (24.8−37.7) | 8.4 (4.3−12.4) | 44.7 (37.1−52.3) | 21.9 (17.9−26.0) | 46.0 (38.6−53.5) | 53.1 (45.8−60.4) |
| Sitting time/day |  |  |  |  |  |  |  |
| ≤ 4 hours | 1595 | 18.1 (16.7−19.5) | 14.4 (13.2−15.7) | 30.8 (28.6−33.1) | 13.9 (12.6−15.3) | 43.1 (40.6−45.7) | 44.1 (41.5−46.8) |
| > 4 hours | 848 | 19.1 (16.8−21.4) | 16.3 (14.8−17.8) | 28.6 (26.1−31.0) | 15.5 (13.2−17.9) | 46.4 (42.7−50.1) | 47.8 (44.6−51.0) |
|  |  |  |  |  |  |  |  |

TG, triglycerides; HDL−C, high−density lipoprotein cholesterol. Data are expressed as number (%, 95%CI). Occupation was categorized as heavy occupation (farmer and manual worker) or none heavy occupation (office clerks, teacher, retired worker, and house worker). Overweight and obesity were defined as BMI ≥ 23 kg/m^2^ and BMI ≥ 25 kg/m^2^. One drink was defined as a 50–ml cup of rice wine at about 30%.

†Age and sex adjustment based on the 2009 Vietnam Population and Housing Census using direct standardization method.

^a^ *P* < 0.05; ^b^ *P* < 0.01; ^c^ *P* < 0.001 by Chisquare test or Fisher exact test: compare metabolic syndrome and its componentts according socio−economic status and lifestyle factors.
