# Supplementary material for: Metabolic syndrome among a middle-aged population in the Red River Delta region of Vietnam
Source: BMC Endocr Disord. 2014 Sep 26;14:77. doi: 10.1186/1472-6823-14-77 (PMC4179436; doi:10.1186/1472-6823-14-77)
Supplement: Additional file 2 — Associated factors of metabolic syndrome in middle-aged population in univariate logistic regression analysis. LDL-C, low-density lipoprotein cholesterol; BMI, body mass index. Educational level was categorized in four groups, by number of years of schooling: elementary level (≤5 years), intermediate level (6–9 years), secondary level (10–12 years), and post–secondary level (>12 years). Occupation was categorized as heavy occupation (farmer and manual worker) or none heavy occupation (office clerks, teacher, retired worker, and house worker). One drink was defined as a 50–ml cup of rice wine at about 30%. High LDL-C was defined when LDL-C ≥ 130 mg/dL (≥3.4 mmol/L); High total cholesterol was defined when total cholesterol ≥ 200 mg/dL (≥5.2 mmol/L). [file 1472-6823-14-77-S2.docx]

**Additional file 2 - Associated factors of metabolic syndrome in middle-aged population in univariate logistic regression analysis**

| **Variable** | **OR (95%CI)** | ***P*** | **Variable** | **OR (95%CI)** | ***P*** |
| --- | --- | --- | --- | --- | --- |
| **Social-economic status** |  |  | **Lifestyle patterns** |  |  |
| Sex |  |  | Residence |  |  |
| Male | 1 |  | Rural | 1 |  |
| Female | 1.26 (1.01-1.58) | 0.040 | Urban | 2.99 (2.10-4.27) | < 0.0001 |
| Age (year) | 1.08 (1.06-1.09) | 0.000 | Alcohol consumption |  |  |
| Marrital status |  |  | None | 1 |  |
| Married | 1 |  | <1 drink/mo | 0.74 (0.45-1.20) | 0.222 |
| Never | 1.87 (0.99-3.51) | 0.051 | ≥ 1 drink/mo to < 1 drink/wk | 1.08 (0.70-1.65) | 0.733 |
| Widowed | 1.31 (0.87-1.98) | 0.203 | 1 drink/wk to ≤ 1 drink/d | 0.65 (0.46-0.92) | 0.015 |
| Others | 1.13 (0.49-2.60) | 0.773 | ≥ 2 drink/d | 0.94 (0.67-1.33) | 0.735 |
| Education level |  |  | Smoking |  |  |
| Elementary | 1 |  | None | 1 |  |
| Intermediate | 0.62 (0.45-0.85) | 0.003 | Current smoker | 0.47 (0.33-0.67) | <0.0001 |
| Secondary | 0.52 (0.34-0.80) | 0.003 | Ex–smoker | 1.22 (0.89-1.69) | 0.218 |
| Post–secondary | 0.91 (0.62-1.33) | 0.617 | Watching TV time/day |  |  |
| Heavy occupation |  |  | ≤ 3 hours | 1 |  |
| Yes | 1 |  | > 3 hours | 1.37 (0.88-2.13) | 0.168 |
| No | 1.57 (1.24-1.98) | < 0.0001 | Sleeping time/day |  |  |
| Income level |  |  | 6–7 hours | 1 |  |
| < 25 percentiles | 1 |  | < 6 hours | 1.12 (0.84-1.50) | 0.426 |
| 25–<50 percentiles | 1.04 (0.77-1.41) | 0.789 | ≥ 8 hours | 0.92 (0.71-1.20) | 0.548 |
| 50–75< percentiles | 1.14 (0.84-1.54) | 0.403 | Sitting time/day |  |  |
| ≥75 percentiles | 1.34 (0.99-1.78) | 0.051 | ≤ 4 hours | 1 |  |
| **Biochemical blood patterns** | |  | > 4 hours | 0.99 (0.80-1.24) | 0.963 |
| High LDL-C |  |  | Siesta time/day (per 15 min) | 1.05 (1.00-1.11) | 0.070 |
| No | 1 |  | **Anthropometric measurements** | |  |
| Yes | 3.12 (2.51-3.88) | < 0.0001 | BMI (kg/m^2^) | 1.30 (1.25-1.36) | < 0.0001 |
| High total cholesterol |  |  | Body fat (%) | 1.11 (1.09-1.13) | < 0.0001 |
| No | 1.0 |  | Hip circumference (cm) | 1.10 (1.08-1.12) | < 0.0001 |
| Yes | 1.42 (1.09-1.85) | 0.009 | Waist-hip ratio (SD=0.07) | 2.27 (1.99-2.60) | < 0.0001 |

LDL-C, Low-density lipoprotein cholesterol; BMI, Body mass index. Educational level was categorized in four groups, by number of years of schooling: elementary level (≤ 5 years), intermediate level (6–9 years), secondary level (10–12 years), and post–secondary level (> 12 years). Occupation was categorized as heavy occupation (farmer and manual worker) or none heavy occupation (office clerks, teacher, retired worker, and house worker). One drink was defined as a 50–ml cup of rice wine at about 30%. High LDL-C was defined as LDL-C ≥ 130 mg/dL (≥ 3.4mmol/L); High total cholesterol was defined as total cholesterol ≥ 200 mg/dL (≥ 5.2 mmol/L).
