# Supplementary material for: Metabolic syndrome among a middle-aged population in the Red River Delta region of Vietnam
Source: BMC Endocr Disord. 2014 Sep 26;14:77. doi: 10.1186/1472-6823-14-77 (PMC4179436; doi:10.1186/1472-6823-14-77)
Supplement: Additional file 3 — The proposal steps for screening metabolic syndrome in community. [file 1472-6823-14-77-S3.docx]

**Additional file 3 -** **The proposal steps for screening metabolic syndrome in community**

| Criteria for first step of screening | Percent population involved in second step of screening | Percent underestimation of prevalence | Percent undetection of MetS cases |
| --- | --- | --- | --- |
| Central obesity | 12.3% | 5.6% | 54% |
| Elevated blood pressure | 29.2 % | 4.4% | 35% |
| Central obesity or Elevated blood pressure | 36.3 % | 2.0 % | 12.3% |
